# Cognitive behavioural therapy and mindfulness for relatives of missing persons: a pilot study

**DOI:** 10.1186/s40814-019-0472-z

**Published:** 2019-07-20

**Authors:** Lonneke I. M. Lenferink, Jos de Keijser, Ineke Wessel, Paul A. Boelen

**Affiliations:** 1 Department of Clinical Psychology and Experimental Psychopathology, Faculty of Behavioural and Social Sciences, University of Groningen, Grote Kruisstraat 2/1, 9712 TS Groningen, the Netherlands; 2 Department of Clinical Psychology, Faculty of Social Sciences, Utrecht University, P.O. Box 80140, 3508 TC Utrecht, the Netherlands; 3 ARQ National Psychotrauma Centre, Nienoord 5, 1112 XE Diemen, the Netherlands

**Keywords:** Disappearance, Ambiguous loss, Missing persons, Treatment, Therapy, Grief, Bereavement, Mindfulness

## Abstract

**Objectives:**

Relatives of long-term missing persons need to deal with uncertainties related to the disappearance. These uncertainties may give rise to ruminative thinking about the causes and consequences of the loss. Focusing on tolerating uncertainties in treatment of relatives of missing persons might foster recovery. Adding mindfulness to cognitive behavioural therapy might serve this aim. The feasibility and potential effectiveness of cognitive behavioural therapy with mindfulness were evaluated in a pilot study. We aimed to detect changes in symptom levels and mindfulness from pre-treatment to 1 week, 12 weeks, and 24 weeks post-treatment.

**Method:**

Dutch adults who experienced the disappearance of a significant other more than 3 months earlier and scored above clinical thresholds for psychological distress were eligible to participate. Participants were recruited from January 2015 to July 2016. Participants in the immediate treatment group started treatment after 1 week after randomization, whereas waiting list controls started the treatment after 12 weeks of waiting. Data from self-report measures as well as clinical diagnostic interviews (tapping persistent complex bereavement disorder, major depressive disorder, and posttraumatic stress disorder) were gathered among 17 relatives of missing persons with elevated symptom levels.

**Results:**

The response rate (31.7%) was low, and dropout rate (47.1%) high. Cognitive behavioural therapy with mindfulness coincided with changes in psychopathology levels (Hedges’ *g* 0.35–1.09) and mindfulness (Hedges’ *g* − 0.10–0.41). Participants completing the treatment were satisfied with treatment quality and reported high treatment compliance.

**Conclusions:**

Because of the limited research about effective treatments for relatives of missing persons and promising results of small and/or uncontrolled trials examining the effect of mindfulness-based treatment to target grief-related complaints, it seems valuable to continue investigating the effects of cognitive behavioural therapy with mindfulness on reducing post-loss psychopathology in future research. However, in order to increase the feasibility of future trials among relatives of missing persons, we recommend collaborating internationally and/or extending duration of recruitment phase, to maximize the sample size.

**Trial registration:**

The Netherlands National Trial Register, NTR4732.

**Electronic supplementary material:**

The online version of this article (10.1186/s40814-019-0472-z) contains supplementary material, which is available to authorized users.

Most people will face the death of someone significant at some point in their lives. Sadness and longing for the deceased are common grief responses. When grief reactions endure and are so intense that they cause significant impairment in daily life, a diagnosis of persistent complex bereavement disorder (PCBD) may be considered. PCBD is included as condition for further study in the fifth Diagnostic and Statistical Manual of Mental Disorders (DSM-5; [1]). PCBD^1^ shows similarities with, yet is distinguishable from major depressive disorder (MDD) and posttraumatic stress disorder (PTSD) [2–4]. About 10% of people exposed to a non-violent loss develop PCBD [5].

Although cognitive behavioural therapy (CBT) is the treatment of choice for loss-related psychopathology [6], only about half of the bereaved people show clinically relevant reductions in PCBD following CBT [7]. Two trials indicate that mindfulness is a useful complementary intervention for bereaved people [8, 9]. For instance, elderly bereaved people with clinically relevant psychopathology levels receiving mindfulness-based CBT (*n* = 12) reported significantly larger reductions in MDD severity from pre-treatment to 5 months post-treatment compared with 18 waiting list controls [8]. In addition, in an uncontrolled trial among a treatment-seeking bereaved sample (*n* = 42), mindfulness-based treatment coincided with significant declines in MDD and PTSD levels from pre- to post-treatment [9].

Compared with literature on emotional distress in bereaved people [6, 10], literature on distress in relatives of missing persons is limited [11]. The scant research in this area suggests that PCBD, MDD, and PTSD are more common following the disappearance of a loved one than after the non-violent death of a loved one. The disappearance of a significant other may be more challenging than separation caused by death, due to the uncertainty about the permanence of the separation [12, 13]. This uncertainty may give rise to ruminative thinking about the whereabouts of the missing person and the circumstances related to the disappearance [14, 15]. At first, perseverative thinking about the disappearance may be helpful in the search of the missing person [16]. As time goes by, perseverative thinking may grow into a maladaptive coping strategy leading to exhaustion, concentration, and sleep problems [15, 17].

Focusing on tolerating uncertainties by adding mindfulness to CBT (henceforth referred to as CBT+M) might be beneficial for relatives of long-term missing persons. Training mindfulness skills teaches people to act with awareness by (1) decentring awareness (i.e. to view inner experience such as thoughts and feelings as temporary and not related to the self), (2) diverting attention toward (rather than away from) painful inner experiences, (3) accepting these inner experiences in a non-judgemental manner, and (4) letting inner experiences pass without reacting [18]. Several trials, in predominantly people with depressive symptoms, have shown that ruminative thinking is an important mechanism of change in mindfulness-based interventions [19].

To the best of our knowledge, only one treatment study among relatives of missing persons has been conducted; this trial included women whose husbands went missing or were killed during the war in Bosnia-Herzegovina. That trial indicated that dialogical exposure group therapy (based on a CBT framework) and supportive group therapy both reduced PTSD and grief (i.e. yielding small to moderate effect sizes) [20]. Yet, the generalizability of the findings to people confronted with a disappearance not related to the war in Bosnia-Herzegovina is limited due to the unique features of this sample (e.g. low levels of literacy, Islamic background). More research is needed to enhance knowledge about the treatment of psychopathology in relatives of missing persons.

We aimed to evaluate the feasibility and potential effectiveness of CBT+M for reducing PCBD, MDD, and PTSD symptoms and enhancing mindfulness among relatives of missing persons with clinically significant psychopathology, using a pilot randomized controlled trial (RCT), comparing CBT+M with a waiting list control condition. A study protocol of this study was published previously [21]. In line with that study protocol, the feasibility of the treatment was examined by reporting (1) participation bias, (2) attrition rate, (3) treatment fidelity, and (4) participants’ evaluations of the treatment. Regarding the preliminary effectiveness of CBT+M, we expected within-group reductions in PCBD, MDD, and PTSD levels and an increase in state mindfulness from pre-treatment to 1 week, 12 weeks, and 24 weeks post-treatment.

In our study protocol [21], we planned to examine three secondary objectives. However, we did not proceed with these analyses, because the final sample size of 17 randomized participants was too small. Firstly, we displayed reductions in percentages in the outcome measures for the treatment and waiting list control group, instead of testing whether changes in symptom and mindfulness levels differed between the groups. Secondly, we visually inspected the patterns of changes and calculated reliable change indices (RCI), instead of statistically testing associations between presumed mechanisms of change (including changes in negative grief cognitions, intrusive memories, rumination, repetitive negative thinking, avoidance behaviours, and self-compassion) and the outcome measures. Thirdly, we were not able to explore session-to-session changes in repetitive negative thinking, intrusive memories, and self-compassion, because too few participants completed measures needed to do so.

## Method

### Participants and procedures

The pilot study is part of a larger Dutch project investigating the impact of the long-term disappearance of a significant other (cf. [16, 21, 22]). Following the definition of the Association of Chief Police Officers [23], a missing person is ‘Anyone whose whereabouts is unknown whatever the circumstances of disappearance. They will be considered missing until located and their well-being or otherwise established’ (p. 15).

Adults who experienced the disappearance of a spouse, family member, or friend more than 3 months earlier were invited to take part in a survey between July 2014 and July 2016 [22, 24–26]. Participants were recruited via (peer) support organizations, a Dutch television show for relatives of missing persons, a website of the research project, and other media attention. Moreover, participants were asked to invite other relatives. The survey was accompanied by a letter that informed participants about a subsequent study designed to evaluate a tailored intervention for relatives of missing persons. Participants who scored above clinical thresholds for PCBD, MDD, and/or PTSD (described below) were potentially eligible for participation in the pilot RCT and received an information letter with details about the treatment and the study.

People who gave written consent for participation in the pilot RCT were interviewed by telephone using the M.I.N.I. Plus version 5.0.0. [27] and the Traumatic Grief Inventory (TGI; [28]). A trained psychologist performed these semi-structured diagnostic interviews aimed at screening for the following inclusion criteria: (1) presence of PCBD, MDD, and/or PTSD; (2) absence of mental retardation; (3) absence of substance abuse; (4) absence of psychotic symptoms; (5) no high risk of suicide; and (6) not concurrently receiving support from a psychologist or psychiatrist. Subsequently, another researcher carried out a blocking randomization procedure. This procedure increases the chance that each condition contains an equal number of participants [29]. Eligible participants were randomly allocated to the immediate treatment group or waiting list control group. Participants allocated to the immediate treatment group started the treatment, whereas the participants of the waiting list control group started the treatment after 12 weeks of waiting. Inclusion in the pilot RCT was possible between January 2015 and July 2016.

**Fig. 1:**
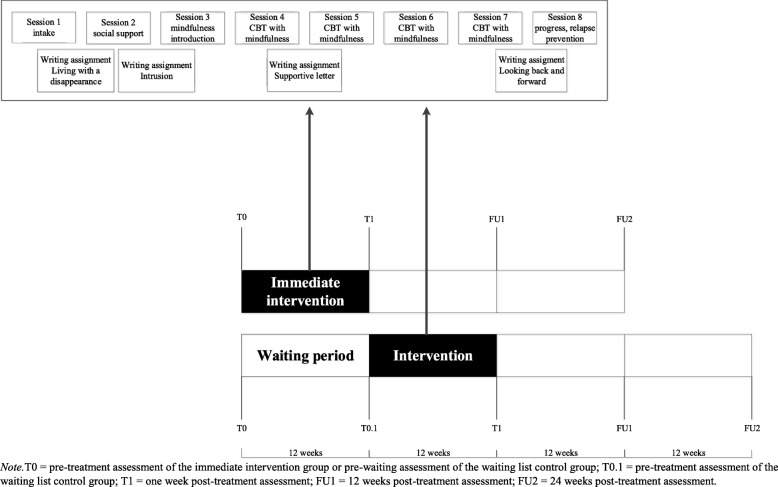
Design of pilot RCT

Participants completed questionnaires before treatment (referred to as T0) and at three time points post-treatment, i.e. after 1 week (referred to as T1), 12 weeks (referred to as FU1), and 24 weeks (referred to as FU2). Participants in the waiting list control group completed an additional questionnaire in the last week of the waiting period (referred to as T0.1) in order to examine between-group effects (treatment vs. waiting). Furthermore, relevant modules of the M.I.N.I (including MDD and PTSD) and the TGI were also administered by an independent psychologist 1 week post-treatment. See Fig. 1 for a schematic display of the design.

### CBT with elements of mindfulness

The manualized treatment consisted of eight individual face-to-face sessions. Drawing from CBT for bereaved individuals [30, 31], the primary aim was to help relatives to change maladaptive cognitions and avoidance behaviours related to the disappearance in session and through homework exercises. Mindfulness and writing exercises were added to CBT as homework assignments. Psycho-education was offered in a treatment manual for clients. Mindfulness exercises were based on mindfulness-based cognitive therapy [32] and were offered on CD-ROM and online [33]. Participants were instructed to practice these exercises at home at least five times a week from session 3 through 8. The aim of mindfulness was to teach participants how to tolerate ambiguity related to the disappearance. Four structured writing exercises served to encourage imaginary exposure, to alter negative cognitions and behaviours and to empower participants. These were derived from internet-based interventions for PCBD [34]. Figure 1 schematically depicts the treatment. The content of the treatment is discussed in more details in our study protocol [21]. For an overview of the themes session-by-session, see Additional file 1. For treatment manuals, including the therapist and client version, see: https://osf.io/af76t/?view_only=18553479967844198e4629ef59346ea6.

Governmentally licensed mental healthcare therapists offered the treatment in the institution where they practised their profession. Therapists were selected from a Dutch nationwide network of therapists who are trained and experienced in treating people with CBT who suffer from grief-related distress after a sudden/violent loss of a significant other. Therapists who had experience with mindfulness in treatment were selected, but they did not have to meet other specific requirements regarding the amount of experience with mindfulness. This network of therapists conducted treatments in prior research from our research group. Therapists received a 1-day training in which the first, second, and fourth author explained the treatment protocol.

### Power analysis

An a priori power analysis showed that 24 participants would be sufficient to find a within-subject difference of a medium effect size in PCBD levels across four measurement occasions (pre-treatment measure, T1, FU1, and FU2) with 80% power and an α of .05. By taking into account a dropout rate of 19% (cf. [6]), we aimed to include 29 participants in total.

### Measures

#### Primary outcome measure

The 19-item Inventory of Complicated Grief (ICG) assessed disturbed grief reactions [35, 36], referred to as PCBD in the current study. Participants were instructed to rate how frequently they experienced each grief reaction (e.g. ‘Ever since he/she has been missing it is hard for me to trust people’) during the preceding month on 5-point scales (0 = ‘never’ to 4 = ‘always’). The ICG has demonstrated adequate psychometric properties. Scores > 25 are indicative of clinically significant grief [36]. Cronbach’s alpha in the current study was .83 at T0.

#### Secondary outcome measures

The 20-item PTSD Checklist for DSM-5 (PCL-5) assessed PTSD levels in accord with the DSM-5 criteria [37, 38]. Participants rated to what extent they experienced each PTSD symptom (e.g. ‘In the past month, how much were you bothered by repeated, disturbing, and unwanted memories of the events that are associated with the disappearance?’) during the preceding month on 5-point scales ranging from 0 = ‘not at all’ to 4 = ‘extremely’. The PCL-5 showed adequate psychometric properties [37]. The provisional cutoff of > 38 [39] or the diagnostic rule of scoring at least a 2 (“moderately”) on at least 1 cluster B item, 1 cluster C item, 2 cluster D items, and 2 cluster E items are indicative of clinically relevant PTSD [1]. Cronbach’s alpha in the current study was .86 at T0.

The 30-item Inventory of Depressive Symptomatology–Self-Report (IDS-SR) assessed MDD levels [40]. Each item consists of a description of a depressive symptom (e.g. ‘Feeling sad’). Participants were instructed to choose one out of four answers (range 0–3) that best described how frequently they experienced the symptom during the preceding week (e.g. ‘I feel sad nearly all of the time’). The IDS-SR showed good psychometric properties [40]. Scores > 13 were indicative of mild depression [41]. Cronbach’s alpha in the current study was .82 at T0.

The 16-item Southampton Mindfulness Questionnaire (SMQ) assessed the ability to respond mindfully to distressing thoughts and images [18, 42]. Participants were instructed to rate their agreement with each item (e.g. ‘Usually when I experience distressing thoughts or images I am able just to notice them without reacting’) on 7-point scales (0 = ‘totally agree’ to 6 = ‘totally disagree’). After reverse coding of some items, higher total scores indicated lower mindfulness in response to distressing thoughts and images related to the disappearance. The SMQ showed good psychometric properties [18]. Cronbach’s alpha in the current study was .73 at T0.

The instructions and/or items of the ICG, PCL-5, and SMQ were adapted to refer to the disappearance. Other measures were used for exploring potential mechanisms of change of treatment. Because we adapted our initial analytic plan, we moved the details and data regarding these measures to Additional file 2.

#### Other measures

In the pre-treatment survey, we asked about the presumed cause of disappearance and belief about the whereabouts of the missing loved one. The presumed cause of disappearance was categorized as follows: voluntary, victim of criminal act, victim of accident, and no (specific) suspicion. Belief about the whereabouts of the missing loved one was categorized as follows: I think (s)he is alive, I doubt whether (s)he is alive, and I think (s)he is not alive. In addition, we asked whether participants had previously sought professional support for dealing with the disappearance. This variable originally consisted of 5 answer categories (1 = yes, I searched for support, but did not find it; 2 = yes, I receive support at the moment; 3 = yes, I received support and I think it was helpful; 4 = yes, I received support, but I think it was unhelpful; and 5 = no, I did not seek support). We dichotomized (i.e. 1 and 5 = no, and 2 to 4 = yes) this variable for the feasibility analyses to avoid small sample sizes in some cells. We also asked ‘Do you have experience with performing mindfulness-exercises?’ with the following answer options: 1 = yes, I practice mindfulness more than once each week; 2 = yes, I practice mindfulness more than once each month; 3 = yes, I practice mindfulness less than once each month; and 4 = no, I don’t practice mindfulness. In the T1 assessment, participants’ perspective on the quality of the treatment was assessed by the following two open-ended questions: (1) what aspects of the treatment are you satisfied with and (2) what aspects of the treatment are you less satisfied with?.

During the administration of the M.I.N.I. and TGI pre- and post-treatment, we asked the participants to rate to what extent they experienced hope that their loved one was still alive on a scale from 1 (‘no hope’) to 10 (‘a lot of hope’; cf. [43]). In addition, we asked participants during the pre-treatment interviews whether they were diagnosed by a psychologist, psychotherapist, or psychiatrist with a mental disorder prior to the disappearance of their loved one with answer options yes or no.

Participants were asked to keep a diary about their experiences with the mindfulness exercises, including questions such as which exercise they conducted at what day and time (henceforth referred to as ‘mindfulness diary’). The therapists were asked to write about the compliance and deviations of the protocol in a diary after each session (henceforth referred to as ‘therapist diary’). This therapist diary included specific items for each session. For instance, (1) did the participant invite a significant other for session two and (2) conduct the homework exercises (e.g. writing exercises)?

### Analyses

#### Feasibility

Series of logistic regression analyses, with one predictor at a time, were performed to examine which background and sociodemographic characteristics and psychopathology levels (i.e. levels of PCBD, MDD, and PTSD) distinguished relatives of missing persons who were willing to receive compared to those who declined professional support. Less than 5% of the data per item was missing, and missing data were therefore imputed with the mean item scores.

With respect to attrition rate, we reported the reasons why participants dropped out of the study, but we were not able to statistically test differences between dropouts (*n* = 8) and completers (*n* = 9) due to the small sample sizes. Regarding treatment fidelity, we reported the (1) number of received treatment sessions, (2) number of days practising mindfulness exercises, (3) whether the participant performed writing exercises, and (4) whether CBT was performed. This was based on screening the therapist diaries, mindfulness diaries, and writing assignments. In addition, during the treatment, adherence to the protocol was monitored by discussing the progress of the treatment with the therapist each month (by telephone or email).

The strengths and improvements of the treatment were described based on the participants’ answers to the two open-ended questions included in the T1 assessment (i.e. ‘What aspects of the treatment are you satisfied with?’ and ‘What aspects of the treatment are you less satisfied with?’). Data of the completers were analysed by the first author who has ample experience in qualitative data analysis, using methods from grounded theory [44]. Accordingly, answers were divided into meaningful units and then labelled with meaningful labels that reflected the content of these units (called subthemes). Overarching major themes across the subthemes were identified (called main themes). In addition to our study protocol, we added two case descriptions to our trial illustrating one successful (i.e. based on RCI scores) and one less successful case of CBT+M (i.e. based on RCI scores), respectively. Both case descriptions were based on information gathered from the therapists. The participants gave written consent for gathering this information. Names and other identifying information were altered in the case descriptions to protect confidentiality.

#### Potential effectiveness

Hedges’ *g* effect sizes correcting for small sample sizes were calculated for comparisons between average symptom levels within participants over time, whereby effect sizes of 0.2 are considered small, 0.5 as moderate, and 0.8 as large [45]. RCIs were calculated for each participant using the following formula ([46], p. 14): RCI$=\frac{X_2-{X}_1}{\mathrm{Sdiff}}$, with *X*_2_ representing a participant’s score at T1, FU1, or FU2; *X*_1_ representing scores at pre-treatment; and Sdiff is calculated using Cronbach’s alpha and standard deviation of the pre-treatment scores. Following prior research (cf. [46]), we considered RCI > 1.96 as clinically significant change. Prevalence rates of PCBD, MDD, and PTSD based on the clinical interviews (including the M.I.N.I. and TGI) prior and post-treatment were summarized. If the participant did not meet diagnostic criteria for PCBD, MDD, and PTSD at post-treatment, this was labelled as ‘in full remission’. Meeting diagnostic criteria for one or two disorders, but fewer disorders post-treatment compared with pre-treatment, was labelled as ‘partly recovery’. No change or increase in number of disorders was labelled as ‘not recovered’.

Contrary to our initial analytic plan [21], we did not report within-subjects and between-subjects statistical analyses (immediate intervention versus waiting list controls) and multiple regression analyses (to test possible mechanisms of change in the treatment), due to the small sample size of the current study. We were also not able to perform the planned analyses with the data that were to be collected each treatment session, because only one participant completed all these measures. Lastly, we did not conduct an intention-to-treat analysis for the within-group comparisons, because of all 8 participants dropping out from the study, 3 did not start the treatment and 5 received only one or two sessions. We did not include these individuals in the analyses, because that was not considered to yield meaningful insights into the preliminary effectiveness of CBT+M [47].

## Results

### Participants

**Fig. 2:**
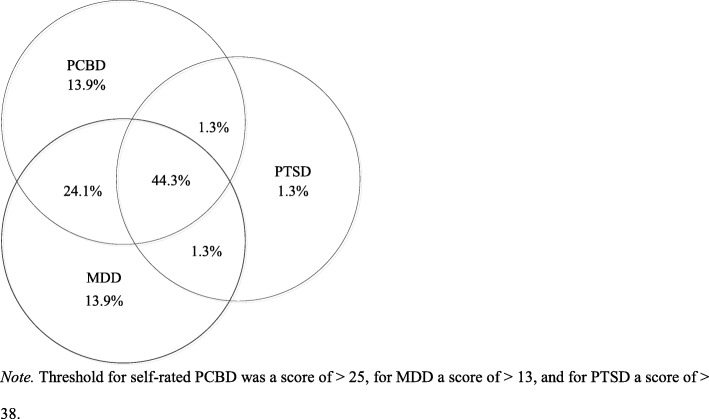
Schematic display of comorbidity between self-rated PCBD, MDD, and PTSD (*n* = 79)

In total, 137 relatives of long-term missing persons participated in the survey. Of them, 66 (48.2%) scored above the threshold of self-rated PCBD, 66 (48.2%) above the threshold for mild MDD, and 38 (27.7%) met provisional criteria for PTSD. In total, 79 (57.7%) passed at least one threshold. Figure 2 depicts comorbidity between clinically relevant levels of self-rated PCBD, MDD, and PTSD among these 79 participants.

**Fig. 3:**
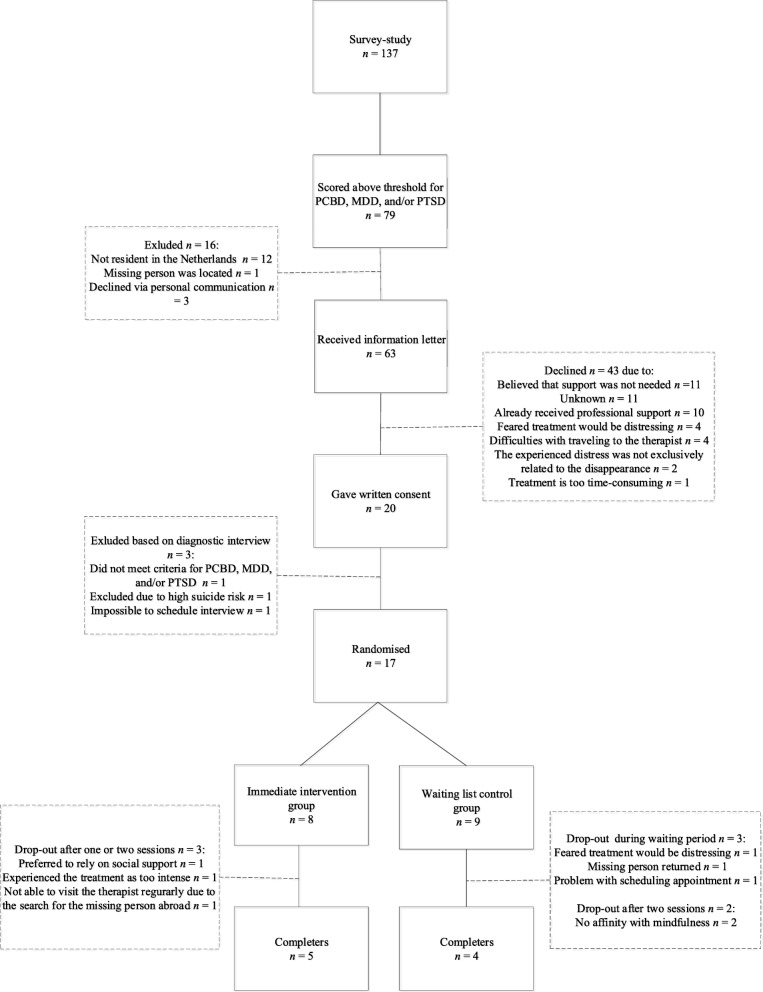
Flowchart of pilot RCT

Sixty-three of these 79 participants were send an invitation letter to participate in the current study (see Fig. 3 for more details). Forty-three potential participants declined. The two primary reasons to decline participation were: 1) I believe that professional support is not needed (25.6%) and 2) I already received professional support (23.3%). Twenty participants signed up for the study, of whom 17 were eligible to participate based on results from the clinical diagnostic interviews (i.e., the M.I.N.I. and TGI; see Fig. 3 for reasons for exclusion of three potential participants). Eight participants were randomly allocated to the immediate intervention group and nine to the waiting list control condition. Five participants of the immediate intervention group and four participants of the waiting list control group completed the treatment (see Fig. 3).

### Feasibility analyses

#### Participation bias

Table 1 shows the characteristics of the people who were eligible to participate in the study but declined (*n* = 43) and people who were eligible and willing to participate in the study (*n* = 20). The logistic regression analyses showed that the latter participants scored significantly higher on MDD and PTSD levels than persons who declined to participate. The two groups did not differ on the other variables.

**Table 1:** Characteristics of people who declined and approved to participate

	People who declined to participate in the study (*n* = 43)	Participants who signed up for the study (*n* = 20)	Odds ratio (95% CI)
Gender (0 = male), *N* (%)	11 (25.6)	6 (30.0)	0.80 (0.25–2.60)
Age, M (SD)	60.62 (13.12)	54.40 (12.79)	0.97 (0.93–1.01)
Educational level (0 = low to moderate), *N* (%)	24 (55.8)	10 (50.0)	1.26 (0.44–3.66)
Kinship (0 = missing person is child/spouse), *N* (%)	22 (51.2)	9 (45.0)	1.28 (0.44–3.71)
Time since disappearance in years, M (SD)	12.68 (14.60)	11.35 (15.78)	0.99 (0.96–1.03)
Fate missing person (0 = criminal act) vs, *N* (%)	13 (30.2)	4 (20.0)	
Voluntarily	12 (27.9)	5 (25.0)	1.35 (0.29–6.26)
Accident	9 (20.9)	5 (25.0)	1.81 (0.38–8.64)
No (specific) presumption	9 (20.9)	6 (30.0)	2.17 (0.47–9.95)
Believe about whereabouts (0 = he/she is dead) vs, *N* (%)	26 (60.5)	9 (45.0)	
doubt whether he/she is alive	11 (25.6)	5 (25.0)	1.31 (0.36–4.82)
he/she is alive	6 (14.0)	6 (30.0)	2.89 (0.74–11.28)
Received previous professional support due to the disappearance (0 = no)	20 (46.5)	10 (50.0)	0.87 (0.30–2.51)
PCBD level, M (SD)	33.53 (11.70)	34.96 (12.01)	1.01 (0.97–1.06)
MDD level, M (SD)	21.81 (11.89)	33.05 (12.46)	1.08 (1.02–1.13)**
PTSD level, M (SD)	27.27 (15.97)	38.19 (13.62)	1.05 (1.01–1.09)*

*PCBD* persistent complex bereavement disorder, *MDD* major depressive disorder, *PTSD* posttraumatic stress disorder, *Exp. (B)* odds ratio, *95% CI* 95% confidence interval

^*^p < .05

^**^p < .01

#### Background characteristics of the participants

Table 2 shows background information about the participants at individual level who were randomized. To safeguard participants’ privacy, some characteristics are not reported in Table 2, but only reported on group level in this paragraph. Of all 17 participants included in the pilot RCT, twelve participants were female (70.6%) and 8 participants (47.1%) had a high educational level. The mean age of the participants was 54.65 (SD = 12.50, range 22 to 71) years. The disappearance took place 11.71 (SD = 16.39) years earlier (range 3 months to 47 years). Four (23.5%) participants had a missing child, four (23.5%) a missing spouse, two (11.8%) a missing parent, six (35.3%) a missing sibling, and one (5.9%) a missing foster child. The presumed reason of the disappearance was in four cases (23.5%) a criminal act (e.g. presumed homicide), four cases (23.5%) a voluntarily disappearance (e.g. run away), three cases (17.6%) an accidental disappearance (e.g. skiing accident), and six persons (35.3%) had no (specific) presumption about the reasons of disappearance.

**Table 2:** Background characteristics of the participants who were randomized (*n* = 17)

Participant ID	Time since disappearance in years^a^	Presumed reason of disappearance	Received previous professional support due to the disappearance?	Diagnosed with a mental disorder prior to the disappearance?	Previous experience with practicing mindfulness	Condition (0 = immediate intervention, 1 = waiting list)
Participants who completed the treatment (*n* = 9)						
ID2	31–40	No specific presumption	Yes	No	No	0
ID3	10–20	Left volutarily	Yes	No	No	1
ID4	1–5	Accident	Yes	No	No	0
ID9	1–5	Criminal act	No	No	No	1
ID11	41–50	No specific presumption	Yes	No	No	0
ID13	41–50	Accident	Yes	No	Yes, > 1 each week	0
ID15	1–5	Left volutarily	Yes	No	No	1
ID16	1–5	Left volutarily	Yes	No	No	0
ID17	1–5	No specific presumption	Yes	No	No	1
Participants who dropped out of treatment (*n* = 8)						
ID1	21–30	Criminal act	No	No	No	1
ID5	< 1	No specific presumption	No	No	No	0
ID6	< 1	No specific presumption	No	No	No	1
ID7	< 1	Criminal act	Yes	No	No	1
ID8	6–10	No specific presumption	No	No	Missing	1
ID10	11–20	Criminal act	No	No	Yes, < 1 each month	1
ID12	< 1	Left volutarily	No	No	Yes, < 1 each month	0
ID14	< 1	Accident	No	No	No	0

^a^Number of years were reported in categories to safeguard participants’ privacy

#### Attrition rate and reasons for dropout

In total, 8 out of 17 participants dropped out (47.1%). Three participants dropped out of the immediate treatment group after receiving one or two sessions. One participant reported to prefer to rely on social support rather than professional support as the disappearance of the significant other occurred less than 1 year before treatment (ID5; i.e. representing participant’s ID number). The second participant reported that the first session of the treatment was too stressful since the disappearance took place less than 1 year earlier (ID12). The third participant was unable to visit the therapist because he/she travelled regularly to search for the missing relative who disappeared abroad less than 1 year earlier (ID14).

Five participants from the waiting list condition dropped out. Three participants dropped out during the waiting period: one because the missing person was located (ID8) and one because he/she worried that the therapy would be too intense (ID10). A third participant repeatedly had difficulties with scheduling appointments with the therapist (ID1). Consequently, ID1 was unable to start treatment within the timeframe of the current study and was therefore considered a dropout. One couple whose relative disappeared less than 1 year earlier received only two sessions (ID6 and ID7) once they eventually started treatment. They were reluctant to receive mindfulness and preferred to continue treatment without mindfulness, and as a result, they could not be included in further analyses. The participants who completed the study all represented a unique missing person case. Due to the small group sizes, we did not statistically test differences between dropouts (*n* = 8) and completers (*n* = 9) in terms of baseline characteristics.

#### Treatment fidelity

Based on the therapist diaries, all nine participants received eight treatment sessions, except for one participant (ID17) who received six sessions. We asked participant ID17 why less than 8 sessions were received. ID17 stated: ‘Since I regularly practice mindfulness, I suffer less from recurrent images and thoughts about the disappearance’. All nine participants conducted the writing assignments. The participants were asked to invite a significant other to discuss social support in session 2; only four participants did so. No other major deviations from the protocol took place, based on monthly communication (by telephone or email) with the therapists. Seven participants gave their consent to collect the mindfulness diaries. Based on these diaries, the participants performed mindfulness exercises during treatment on average on 25 days (range 11 to 49 days). None of the participants received additional support from a psychologist, psychotherapist, or psychiatrist after completion of the treatment (assessed at 12 weeks and 24 weeks post-treatment), except for one participant (ID9).

### Two case descriptions

#### Successful treatment

Eva (ID2; fictional name) was almost 40 years old when her brother was travelling around the world for over 2 years. One day she lost contact with her brother who was still abroad. After repeated searches, they only found his bike. Due to her brother’s disappearance, her family of origin was disrupted. Eva’s mother was so torn apart by the disappearance that she died of a broken heart, according to Eva. Eva struggled with her emotions regarding the loss of her brother and mother and was unable to find emotional support from her family of origin, but also from her own husband and children. She became severely depressed and was institutionalized for her depression. Thirty-three years later when she signed up for the study, her brother was still missing. She experienced weekly intrusions regarding her brother’s disappearance.

Based on the diagnostic interview before treatment, Eva met criteria for PCBD, MDD, and PTSD. One week post-treatment, Eva no longer met any of the diagnostic criteria. At the start of the treatment, Eva felt lonely and cried when she talked about her missing brother. During her childhood, Eva’s brother played an important role in her life. Their parents were traditional in terms of that Eva was expected to become a good wife and mom instead of going to school and work. Eva’s brother was expected to become a priest. Eva and her brother supported each other to make their own choices in life. Eva’s brother fled from the life that was planned for him by his parents by travelling the world. In treatment and by conducting the writing assignments, Eva realized how important her brother was to her and how important it was for Eva to speak out freely about her thoughts and feelings. She had not only lost her brother, but also her support to stand up for herself. The therapist emphasized that it was his choice to leave, which reduced Eva’s guilt feelings. Her intrusions were replaced by positive memories regarding her brother. The mindfulness exercises were helpful to Eva; they calmed her down and helped her to confront and tolerate the sadness when thinking about her brother, and she was able to enjoy the little things in life more. At the end of treatment, she felt more capable of tolerating the sadness surrounded by the disappearance and was determined to continue to compensate the sadness by focusing more on what is important to her.

#### Less successful treatment

About 4 years earlier, Lucy (ID9; fictional name) was a single mother who took her six children on holiday to South Africa. Her oldest child Mary was 16 years old at the time, born in South Africa, but raised by her foster mother Lucy in the Netherlands. During their stay in a hotel, Mary disappeared at nighttime. After days of searching, Lucy received a phone call by Mary’s biological mother who told her that she took Mary and that Lucy would never see her again.

Lucy expressed that she was hesitant to start treatment, because she stated that she could cope quite well with the disappearance. When she started to talk and write about the disappearance in treatment, strong feelings of guilt arose from thoughts as ‘If Mary had stayed in my room, it might would not have happened’. These thoughts coincided with intrusive images about the night of Mary’s disappearance. Although Mary conducted assignments on challenging her unhelpful thoughts (e.g. ‘I failed as a mother’), she was not convinced that this was beneficial. She argued that she was already aware of her own cognitive pitfalls. She thought that the writing and mindfulness exercises suited her better, because it helped her to get in touch with her emotions. These exercises were emotionally intense for Lucy, because she was afraid that she would lose control over her emotions. From when she was little, she taught herself to control her emotions, because she did not want to turn out like her mother. Her mother has always been emotionally unstable and was therefore not able to take care of her and her brothers. Similar to when she was younger, Lucy always felt the urge to take care of others. Mary’s disappearance did not only trigger Lucy’s anxiety to fail, it also fueled her strong sense of responsibility. For instance, she was worried that her other children were traumatized by the disappearance. Imaginary exposure assignments were conducted to expose Lucy to challenging emotional situations. Lucy found this helpful but also stressful. Overall, the treatment was perceived as insightful, but it ‘cut open previous wounds’ related to adversity in Lucy’s childhood that was triggered by the disappearance Lucy said, and therefore, she received more therapy sessions afterwards.

Notably, after treatment, Lucy no longer met the criteria for PCBD and MDD. However, her scores on the questionnaires at T1, FU1, and FU2 indicated that her psychopathology levels increased compared with pre-treatment. Lucy received additional support from another therapist between T1 and FU2, which may explain the increase in psychopathology levels post-treatment. An explanation for the deviation between survey and interview scores 1 week post-treatment is that Lucy realized after treatment that her complaints were more attributable to non-disappearance-related issues; she therefore may have reported similar PCBD levels at the pre-treatment survey and T1, but during the interview post-treatment, she emphasized that her primary complaints were not grief-specific (resulting in absence of PCBD). In contrast to the MDD questionnaire, we specifically asked in the interview if MDD symptoms were attributable to the disappearance. This may explain why her MDD levels in the survey increased, but MDD related to the disappearance was absent during the interview.

### Strengths and improvements of the treatment from the participants’ perspective

Based on the qualitative analysis of the answers to the first open-ended question (i.e., ‘What aspects of the treatment are you satisfied with?’), all participants mentioned at least one aspect of the treatment that they appreciated. Six participants were satisfied with the client-therapist relationship (ID2, ID3, ID11, ID13, ID15, ID17). They reported that they felt connected with the therapist and described the therapeutic atmosphere as safe and supportive (‘I felt safe and supported during the treatment. There was all the attention for the grief.’ ID15). Five participants wrote that the mindfulness exercises were a strong element of the treatment (e.g., ID3, ID9, ID11, ID13, ‘Mindfulness is a pleasant method for me to keep myself balanced. I will continue it at fixed times’ ID17), three participants were satisfied with the writing exercises (ID2, ID9, ID13), and two participants mentioned the CBT part as beneficial (ID3, ID15).

Based on the qualitative analysis of the answers to the second open-ended question (i.e., ‘What aspects of the treatment are you less satisfied with?’), four participants wrote that they did not have suggestions for improvement. Five participants gave the following suggestions for improvement. Four participants mentioned aspects of the content of the treatment they did not appreciate. Three participants were less satisfied with the treatment protocol: one mentioned that he/she would like to attend to more than eight sessions (ID3), another participant (ID13) suggested to use fewer assessments (not clinical interview and surveys together), and one participant reported that the protocol was too strict (‘It was too much according the protocol, it therefore felt impersonal.’ ID4). Two participants were not optimistic about the use of mindfulness (ID15, ID16) of which one mentioned that trauma-focused therapy would be more suitable (“I wonder if the traumatic character of a disappearance is sufficiently tackled with mindfulness. I think something like ‘trauma-treatment’ is needed.” ID16). Another participant felt uncomfortable about the amount of homework (ID4), and one participant (ID9) mentioned to prefer to focus more on other issues, not solely related to the disappearance.

### Self-rated PCBD, MDD, PTSD, and mindfulness levels from pre- to post-treatment

Because of the small sample size, we did not report tests to examine within-subjects effects. Table 3 shows the observed individual and mean scores for PCBD, MDD, PTSD, and mindfulness for all nine completers. All participants reported a decline in PCBD, MDD, and/or PTSD levels post-treatment except for one participant (ID9) who reported an increase in psychopathology levels and one participant (ID15) who reported somewhat stable psychopathology levels over time.

**Table 3:** Self-report individual and mean scores of PCBD, MDD, PTSD, and mindfulness before and after treatment and interview-based prevalence rates for the completers (*n* = 9)

Self-report	Measurement occasion	ID numbers									Effect size compared with pre-treatment	
		ID2	ID3	ID4	ID9	ID11	ID13	ID15	ID16	ID17	Mean (SD)	Hedges’ *g*
PCBD scores	Pre-treatment	26	19	22	17	33	32	38	55	25	29.67 (11.70)	–
	T1	13*	21	19	21	19*	27	38	47	25	25.56 (10.62)	0.35
	FU1	6*	26	11	25	23	16*	37	55	16	23.89 (14.82)	0.41
	FU2	7*	19	11	23	19*	22	35	51	11*	22.00 (13.67)	0.57
MDD scores	Pre-treatment	40	36	37	12	44	28	22	48	12	31.00 (13.29)	–
	T1	19*	24	18*	22	25*	24	15	29*	8	20.47 (6.26)	0.97
	FU1	5*	28	16*	17	17*	11*	26	32*	11	18.11 (8.89)	1.09
	FU2	12*	26	13*	28*	19*	19	18	28*	5	18.74 (7.79)	1.07
PTSD scores	Pre-treatment	33	22	32	15	58	52	21	55	12	33.33 (17.68)	–
	T1	18*	21	23	14	30*	26*	17	45	9	22.56 (10.50)	0.71
	FU1	15*	17	12*	22	17*	10*	31	46	4	19.33 (12.55)	0.87
	FU2	5*	18	11*	39*	23*	17*	24	42*	2	20.11 (13.75)	0.80
Mindfulness scores	Pre-treatment	43	59	25	23	48	63	46	53	43	44.78 (13.63)	–
	T1	34	50	57*	48*	48	58	25*	57	38	46.11 (11.50)	− 0.10
	FU1	32	61	29	37	48	51	51	56	26	43.44 (12.68)	0.10
	FU2	4*	51	26	43*	47	56	43	54	18*	38.00 (17.96)	0.41
Diagnostic interviews											% met criteria	
PCBD diagnosis	Pre-treatment	Yes	Yes	Yes	Yes	Yes	Yes	Yes	Yes	Yes	100	–
	Post-treatment	No	No	No	No	Yes	No	Yes	Yes	Yes	44.4	–
MDD diagnosis	Pre-treatment	Yes	Yes	Yes	Yes	Yes	Yes	Yes	Yes	Yes	100	–
	Post-treatment	No	Yes	No	No	Yes	No	Yes	No	No	33.3	–
PTSD diagnosis	Pre-treatment	Yes	No	No	No	Yes	Yes	No	Yes	Yes	55.6	–
	Post-treatment	No	No	No	No	Yes	No	No	No	No	11.1	–
Extent of hope	Pre-treatment	1	8	2	8	5	1	10	10	10	–	–
	Post-treatment	1	5	1	5	5	1	9	10	8	–	–

The 1 week pre-treatment assessment consists of T0 data of the immediate intervention group and T0.1 data of the waiting list control condition. *T1* 1 week post-treatment assessment, *FU1* 12 weeks post-treatment assessment, *FU2* 24 weeks post-treatment assessment, *PCBD* persistent complex bereavement disorder, *MDD* major depressive disorder, *PTSD* posttraumatic stress disorder. Column 3 to 11 represent individual scores, and in case the score at T1, FU1, and FU2 significantly (*p* < .05) reliable differed from the pre-treatment score, based on the reliable change index, it was marked with “*”

*p < .05

For PCBD, symptom levels declined on average between pre-treatment to T1 (Hedges’ *g* = 0.35), FU1 (Hedges’ *g* = 0.41), and FU2 (Hedges’ *g* = 0.57). Based on the individual RCI, two participants (22.2%) reported clinically significant reductions in PCBD levels from pre-treatment to T1 and FU1. Three participants (33.3%) reported clinically significant reductions in PCBD levels from pre-treatment to FU2.

For MDD, symptoms decreased on average from pre-treatment to T1 (Hedges’ *g* = 0.97), to FU1 (Hedges’ *g* = 1.09), and to FU2 (Hedges’ *g* = 1.07). Based on the individual RCI, four participants (44.4%), five participants (55.6%), and four participants (44.4%) reported clinically significant reductions in MDD levels from pre-treatment to T1, FU1, and FU2, respectively. One participant (11.1%) reported clinically significant increase in MDD levels from pre-treatment to FU2.

For PTSD, symptoms decreased on average from pre-treatment to T1 (Hedges’ *g* = 0.71), to FU1 (Hedges’ *g* = 0.87), and to FU2 (Hedges’ *g* = 0.80). Based on the individual RCI, three participants (33.3%), four participants (44.4%), and five participants (55.6%) reported clinically significant reductions in PTSD levels from pre-treatment to T1, FU1, and FU2, respectively. One participant (11.1%) reported clinically significant deterioration in PTSD levels from pre-treatment to FU2.

Compared with pre-treatment mindfulness levels, the mindfulness levels increased at T1 (Hedges’ *g* = − 0.10) but decreased at FU1 (Hedges’ *g* = 0.10) and FU2 (Hedges’ *g* = 0.41) on average. Based on the individual RCI, one participant (11.1%), zero (0.0%), and two participants (44.4%) reported clinically significant improvements in mindfulness (indicated by lower mindfulness levels) from pre-treatment to T1, FU1, and FU2, respectively. Two participants (22.2%) reported clinically significant decrease (indication for less mindfulness) from pre-treatment to T1, and one participant (11.1%) clinically significant decrease in mindfulness levels from pre-treatment to FU2.

### Interview-based PCBD, MDD, and PTSD prevalence rates from pre- to post-treatment

Based on the diagnostic interview, all participants met criteria for PCBD and MDD before treatment. Five out of nine participants (55.6%) met criteria for PTSD before treatment. Four participants (ID2, ID4, ID9, ID13) were in full remission post-treatment, three partly recovered (ID3, ID16, ID17), and two did not recover (ID11, ID15). See Table 3 for more details.

Before and after treatment, we asked the participants about their hope that the missing relative was still alive. The extent of hope seemed to remain quite stable prior and post-treatment (see the last two rows in Table 3). Those who had the least hope that their missing relative was still alive before treatment (ID2, ID4, ID13) were those who were in full remission post-treatment.

### Reductions in symptom levels between the immediate intervention and waiting list control condition

The sample sizes of the two conditions (immediate intervention and waiting list control condition) were too small to statistically test within-subjects and between-subjects treatment effects. Instead, we displayed the reductions (in percentages) in the outcome measures in Additional file 3. In short, the participants in the immediate intervention group (*n* = 5) had at least twice as large reduction in PCBD, MDD, and PTSD levels on average from baseline to post-treatment compared with difference in scores from baseline to post-waiting period of the waiting list controls (*n* = 4).

## Discussion

This study evaluated the feasibility and potential effectiveness of CBT+M in terms of reductions of PCBD, MDD, and PTSD, and enhancement of mindfulness among relatives of missing persons. Given that the current study is, to the best of our knowledge, the first trial examining the effects of a treatment solely for relatives of missing persons, we examined the feasibility and potential effectiveness of a treatment specifically tailored to this unique population. We adapted a grief-specific CBT protocol ([30, 31] by adding elements of mindfulness (derived from mindfulness-based cognitive therapy [32])) and writing assignments (derived from internet-based grief therapy [34]).

The relative high numbers of people scoring above clinical thresholds for psychopathology found in our sample of 137 relatives of missing persons suggest that there is a need for professional support for this unique population. To illustrate this, the rates of clinically relevant self-rated levels of PCBD (48.2%), MDD (48.2%), and PTSD (27.7%) are higher in the sample of people confronted with the disappearance of a loved one, on average 15 years earlier, than rates found in people confronted with a non-violent loss in the past 6 months using comparable instruments and cutoffs [4, 48]. While the rates found in the current study may not be representative, because of our self-selected sample, previous studies also showed high rates of clinically relevant psychopathology levels among people confronted with the disappearance of a loved one [11]. It is remarkable that about half of these relatives of missing persons with elevated psychopathology levels received previous professional support related to the disappearance, pointing to the need of optimizing treatment for relatives of missing persons.

Those who scored above the threshold for PCBD, MDD, and/or PTSD were invited to take part in this pilot study, but 68.3% declined. They thought it was unnecessary or reported that they already received professional support. Furthermore, those who declined reported lower MDD and PTSD levels than those who signed up for the study. These findings indicate that our inclusion criteria may have been too liberal (e.g. mild depression levels instead of severe levels). In general, it is difficult to include participants in trials examining loss-related psychopathology (considering the sample sizes of conditions in grief trials vary from 11 to 101 (see for an overview [49])). Obtaining a large sample of relatives of missing persons, in a small country such as the Netherlands, in which the occurrence of a disappearance is rare [50], would take many years. The limited response rate could also partly be explained by the use of an outreach recruitment strategy. Recruitment of hard-to-reach or rare populations, such as relatives of missing persons, is challenging, and we therefore actively recruited participants who did not initially seek treatment [51].

Our dropout rate from the treatment of 43.8% (i.e. when not taking into account the participant whose missing loved one returned) was considerably higher than the anticipated 19.0% based on previous studies evaluating CBT for bereaved people [6]. It should be noted that most people who discontinued treatment experienced the disappearance in the preceding year and were still actively searching for the missing person or thought that the therapy was too intense. It therefore seems recommendable to offer treatment at least 1 year post-disappearance, which is also in line with the time criterion for PCBD in the DSM-5 [1] and previous trials among people confronted with a loss, for instance [52]. One couple discontinued treatment after two sessions, because they expected that mindfulness was not helpful to them. This could have been prevented by providing more detailed information about the content of the treatment before signing up for the treatment. For instance, in our information letter, we did not explicitly refer to the use of mindfulness in treatment.

With regard to the feasibility of the treatment protocol, no major deviations were reported, except that not all participants were able to invite a significant other to the treatment. Only one participant reported that he/she preferred more therapy sessions, indicating that the other participants thought eight sessions were sufficient, although eight sessions are relatively few compared with other grief treatments [49]. All participants conducted the writing and mindfulness exercises. Overall, participants were satisfied with the content and implementation of the treatment, but some were less satisfied with the amount of homework (including mindfulness exercises), number of assessments, and the strictness of the protocol.

Concerning the potential effectiveness of the treatment, our primary aim was to examine whether participants could benefit from the treatment. On average, the expected patterns of reductions in PCBD, MDD, and PTSD from pre-treatment to 1 week, 12 weeks, and 24 weeks post-treatment were observed. More specifically, for PCBD, small to moderate effect sizes were found; for MDD, large effect sizes; and for PTSD, moderate to large effect sizes at 1 week, 12 weeks, and 24 weeks post-treatment compared with pre-treatment. Six out of nine participants reported significant reliable reductions in PCBD, MDD, and/or PTSD levels. One participant reported increases in psychopathology after treatment. Because this participant is the only participant who received additional support following the treatment, it is unknown whether this increase is due to CBT+M. Changes in PCBD, MDD, and PTSD levels were summarized for the immediate intervention and waiting list control condition to give an indication of the potential effectiveness of CBT+M compared with natural remission. These findings suggest that the intervention contributed to the alleviation of psychopathology levels.

The clinical interviews, including the M.I.N.I. and TGI, showed similar results. Overall prevalence rates of psychopathology post-treatment substantially declined compared with pre-treatment prevalence rates. We also assessed the experienced extent of hope that the missing relative was still alive pre- and post-treatment during the interviews. Because the treatment was focused on tolerating ambiguity instead of adapting it, it is not surprising that the levels of hope seem to remain stable in treatment. Noteworthy, those who had no hope that their loved one was still alive seem to benefit most from the treatment. This finding is in line with previous research indicating that more hope among relatives of missing persons is related to elevated psychopathology levels [43].

Unexpectedly, on average, the mindfulness levels seem to increase (representing less mindfulness) from pre-treatment to 1 week post-treatment. This increase on average is due to two people (ID4 and ID9) reporting a reliable increase in mindfulness levels post-treatment, whereas for the other people, mindfulness remained stable or decreased (representing an improvement in mindfulness). Participant ID4 was also the one who stated that he/she found the protocol too strict and was not satisfied with the amount of homework. This dissatisfaction may have led to less practice of mindfulness, which has been related to lower mindfulness levels in previous research [53]. Participant ID9 reported that CBT+M cut open old wounds related to childhood adversity, not to the disappearance, and she continued treatment after eight sessions. This could be interpreted as if the treatment gave rise to negative thoughts, which she found difficult to tolerate, which may explain the increase in mindfulness levels. Previous research has found that mindfulness is one of the most important mechanisms of change in mindfulness-based interventions [19]. To enhance our understanding of how mindfulness-based grief treatments work, it would be worthwhile for future research to examine to what extent mindfulness, but also other potential mediators, such as ruminative thinking and self-compassion [19], mediates the therapeutic effects.

### Limitations and recommendations

Several limitations should be taken into account. First and foremost, the sample size was too small to draw any firm conclusions about the effectiveness of CBT+M. One way of overcoming recruitment difficulties is to collaborate internationally and/or extend the duration of the recruitment phase. The small sample size necessitated us to remove our secondary objectives from our initial analytic plan [21]. For instance, we were unable to test statistical differences between post-treatment/post-waiting psychopathology levels of the immediate intervention group and waiting list control condition. Furthermore, even if we were able to recruit sufficient participants, our design was limited because we included a waiting list control group instead of an active control group. The two previous trials that examined the effects of mindfulness-based treatment for people confronted with a loss did not include a control group [9] or used a waiting list control group [8]. Consequently, the additional effect of integrating mindfulness in the treatment of loss-related distress remains to be studied. Studies comparing the effects of CBT only with CBT+M might enhance our knowledge about the efficacy of mindfulness for the treatment of people confronted with a loss.

We were also not able to collect sufficient data at each treatment session for examining potential mechanisms of change, because if collected it contained too much missing data or these data were not collected because it was too time-consuming according to the therapists. Instead of collecting these data at the start of each treatment session, using the therapist as test instructor, it might be more successful to collect these data before the start of the treatment session, preferably by the researcher.

Due to the small sample size and high dropout rate, we only reported the scores of the completers, which may overestimate the preliminary effectiveness [47]. Future studies with sufficient sample sizes should include participants in the analyses who dropped out of the treatment. This might yield a more accurate estimate of the efficacy of a treatment in clinical practice, because discontinuing treatment is also likely to occur in daily practice [47].

We developed and used a treatment protocol of CBT+M that was based on, but not directly comparable to, CBT for PCBD [30, 31] and MBCT for recurrent depression [32]. Using one of the original protocols in our study could have increased the comparability of treatment effects between study samples; however, we chose to combine these protocols for three reasons. Firstly, MBCT consists of 2-h weekly group sessions, which we anticipated was not feasible to organize, taking into account that a long-term disappearance is rare in the Netherlands. Recruiting sufficient participants throughout the Netherlands and offering group sessions at one location could heighten barriers to care. Secondly, MBCT is not grief-specific. Prior research has shown that grief-specific CBT is most effective for treating distressed people confronted with loss [49]. Thirdly, as explained in our study protocol [21], we added mindfulness exercises to grief-specific CBT to focus more on tolerating uncertainty related to the loss (with mindfulness), apart from confrontation with irreversibility of the loss (in grief-specific CBT).

Because we focused on relatives of missing persons in the Netherlands and consequently did not include relatives of people who went missing in war or due to political repression abroad, it is unknown to what extent these recommendations apply to people exposed to the disappearance of a significant other in armed conflict. Given the growing number of refugees and people living in conflict areas who are confronted with the disappearance of a significant other [54, 55], it might be fruitful to explore to what extent (parts of) our protocol could be effectively implemented in this much larger group of relatives of missing persons. People exposed to the disappearance of a significant other in armed conflict are likely also exposed to trauma and (multiple) loss [54]. Current treatment approaches for refugees, such as narrative exposure treatment, are predominantly focused on reducing PTSD levels [56], whereas it is unknown to what extent these treatments are effective for reducing PCBD levels [57]. Adding modules to existing treatments for refugees, for instance, CBT+M to target grief-related distress, might give first insights into the effectiveness of such treatments.

## Conclusions

Notwithstanding these limitations, the results of this study are not merely disappointing. CBT+M seems feasible and seems to yield improvements in psychopathology levels based on self-report questionnaires and diagnostic interviews for most, but not all participants. Because of the (1) limited research about effective treatments for relatives of missing persons, (2) elevated risk for psychopathology in relatives of missing persons, and (3) promising results of small and/or uncontrolled trials examining the effect of mindfulness-based treatment to target grief-related complaints, it seems valuable to continue investigating the effects of CBT+M on reducing post-loss psychopathology in future research.

## Additional files

Additional file 1: Supplementary material A. (DOCX 15 kb)

Additional file 2: Supplementary material B. (DOCX 20 kb)

Additional file 3: Supplementary material C. (DOCX 38 kb)

## Data Availability

The data used in the current study are presented in the manuscript (Table 3 and Additional file 2). Questionnaires used in this study are not available due to copyrights. Treatment materials can be found on https://osf.io/af76t/?view_only=18553479967844198e4629ef59346ea6.

## Abbreviations

CBT: Cognitive behavioural therapy
CBT+M: Cognitive behavioural therapy with elements of mindfulness
DAAPGQ: Depressive and Anxious Avoidance in Prolonged Grief Questionnaire
DSM-5: The fifth edition of the Diagnostic and Statistical Manual of Mental Disorders
FU1: 12 weeks post-treatment assessment
FU2: 24 weeks post-treatment assessment
GCQ: Grief Cognitions Questionnaire
IDS-SR: Inventory of Depressive Symptomatology–Self-Report
MDD: Major depressive disorder
PCBD: Persistent complex bereavement disorder
PCL-5: PTSD Checklist for DSM-5
PTQ: Perseverative Thinking Questionnaire
PTSD: Posttraumatic stress disorder
RCI: Reliable change index
RCT: Randomized controlled trial
RRS: Ruminative Response Scale
SCS: Self-Compassion Scale
SMQ: Southampton Mindfulness Questionnaire
T0: Baseline assessment
T0.1: Post-waiting period assessment
T1: 1 week post-treatment assessment
TGI: Traumatic Grief Inventory
TMQ: Trauma Memory Questionnaire

## Footnotes

1 The term persistent complex bereavement disorder (PCBD) is used throughout this article to refer to persistent severe grief reactions that cause impairment in functioning. In the literature, other terminologies including ‘complicated grief’, ‘traumatic grief’, or ‘prolonged grief’ have been used to denote disturbed grief reactions.
