# Supplementary material for: Cognitive behavioural therapy and mindfulness for relatives of missing persons: a pilot study
Source: Pilot Feasibility Stud. 2019 Jul 20;5:93. doi: 10.1186/s40814-019-0472-z (PMC6642737; doi:10.1186/s40814-019-0472-z)
Supplement: Supplementary file 1 — Supplementary material A. (DOCX 15 kb) [file 40814_2019_472_MOESM1_ESM.docx]

**Additional file 1**

**Table 1.** Content of the treatment session-per-session

| **Content of the session** | **Brief description** | **Instruction *at start* of session** | **Homework assignments *after* session** |
| --- | --- | --- | --- |
| Session 1: Introduction, identification of complaints, and psychoeducation | Therapist asks about the disappearance, identifies psychological complaints, and explains the rationale of treatment and handles out the treatment manual. | Completing brief questionnaire | Treatment manual: Reading of chapter 1 to 3, and paragraph 4.5  Writing assignment 1 (2 x 45 min. per assignment) |
| Session 2: Providing and receiving social support | In the second session the client is asked to invite a significant other who is willing to offer social support. | Completing brief questionnaire  Reflections on writing assignment 1 | Treatment manual: Reading of chapter 3 and 4  Writing assignment 2 (2 x 45 min. per assignment) |
| Session 3: Introducing mindfulness | Introducing mindfulness and encouraging mindfulness practices at home. | Completing brief questionnaire  Reflections on writing assignment 1 | Treatment manual: Reading of chapter 4  Practicing mindfulness exercises 5 days per week  Updating mindfulness diary |
| Session 4: Identifying thought patterns and reducing repetitive negative thoughts by mindfulness-exercises | The client learns how thought patterns are related to his/her coping with the disappearance. The therapist offers a framework for identifying and observing thought patterns and challenging unhelpful thought patterns. Possible intrusive memories/images are targeted with exposure-assignments. | Completing brief questionnaire  Reflections on mindfulness-diary | Writing assignment 3 (2 x 45 min. per assignment)  Treatment manual: Reading of chapter 4  Practicing mindfulness exercises 5 days per week  Updating mindfulness diary |
| Session 5: see description session 4 | see description session 4 | Completing brief questionnaire  Reflections on writing assignment 3  Reflections on mindfulness-diary | Treatment manual: Reading of chapter 4  Practicing mindfulness exercises 5 days per week  Updating mindfulness diary |
| Session 6: see description session 4 | see description session 4 | Completing brief questionnaire  Reflections on mindfulness-diary | Practicing mindfulness exercises 5 days per week  Updating mindfulness diary |
| Session 7: see description session 4 | see description session 4 | Completing brief questionnaire  Reflections on mindfulness-diary | Writing assignment 4 (2 x 45 min. per assignment)  Practicing mindfulness exercises 5 days per week  Updating mindfulness diary |
| Session 8 | Appointments are made about ending or continuing treatment and relapse is discussed | Completing brief questionnaire  Reflections on writing assignment 4  Reflections on mindfulness-diary |  |
