# Supplementary material for: Cognitive behavioural therapy and mindfulness for relatives of missing persons: a pilot study
Source: Pilot Feasibility Stud. 2019 Jul 20;5:93. doi: 10.1186/s40814-019-0472-z (PMC6642737; doi:10.1186/s40814-019-0472-z)
Supplement: Supplementary file 2 — Supplementary material B. (DOCX 20 kb) [file 40814_2019_472_MOESM2_ESM.docx]

**Additional file 2**

*Measures*

The 24-item Dutch version of the Self-Compassion Scale (SCS) assessed levels of self-compassion [58]. The participants rated how often they behave in the stated manner on 7-point scales with anchors 1 = “almost never” and 7 = “almost always” (e.g., “I try to see my failings as part of the human condition”). Higher scores indicated higher levels of self-compassion. The SCS showed adequate psychometric properties [1]. Cronbach’s alpha in the current study was .89 at T0.

The 8-item subscale “Intrusion and disorganization” of the Dutch version of the Trauma Memory Questionnaire (TMQ; [2]), assessed levels of intrusive memories related to the disappearance (e.g., “I experience strong emotions when remembering the disappearance”). The participants rated their agreement with each item on 5-point scales ranging from 1(“not at all”) to 5 (“very strongly”). The TMQ showed adequate psychometric properties [2, 3]. Cronbach’s alpha in the current study was .82 at T0.

Nineteen items, representing four subscales, of the Grief Cognitions Questionnaire (GCQ; [4]), assessed levels of negative cognitions related to the disappearance. These items included negative beliefs about the self (six items, e.g., “I see myself as a weak person since he/she disappeared”), life (four items, e.g., “My life is useless since he/she has been missing”), the future (five items, e.g., “I don’t expect that I will feel better in the future”), and one’s own grief reactions (four items, e.g., “If I let go of my emotions, I will go crazy”). Participants rated their agreement with each item on 6-point scales with anchors 0 = “disagree strongly” and 5 = “agree strongly”. The GCQ showed adequate psychometric properties [4]. Cronbach’s alpha in the current study was .92 at T0.

The 9-item Depressive and Anxious Avoidance in Prolonged Grief Questionnaire (DAAPGQ) assessed avoidance behaviour [5]. The DAAPGQ represents depressive avoidance (five items; e.g., “I develop very few new activities since he/she has been missing, because I’m unable to do so.”) and anxious avoidance (four items; e.g., “I avoid to dwell on painful thoughts and memories connected to his/her disappearance.”) Participants rated each item on 6-point scales with anchors 0 = “not at all true for me” to 5 = “completely true for me”. The DAAPGQ has adequate psychometric properties [5]. Cronbach’s alpha in the current study was .77 at T0.

The 15-item Perseverative Thinking Questionnaire (PTQ) assessed levels of disappearance-related repetitive negative thinking (e.g., “I keep asking myself questions without finding an answer”; [6, 7]). Participants rated each item on 5-point scales ranging from 1 = “never” to 5 = “almost always”. The PTQ has adequate psychometric properties [6, 7]. Cronbach’s alpha in the current study was .95 at T0.

The 5-item brooding subscale of the Ruminative Response Scale (RRS) assessed levels of ruminative thinking about a depressed mood (e.g., “I think “What am I doing to deserve this?”; [8, 9]). Participants rated each item on 4-point scales ranging from 1 = “almost never” to 4 “almost always”. The RRS has adequate psychometric qualities [8, 9]. Cronbach’s alpha in the current study was .23 at T0.

We adapted the wording referring to “death” to “disappearance” in the TMQ, GCQ, and DAAPGQ.

*Results*

Table 1 shows the individual and mean scores on the SCS, TMQ, GCQ, DAAPGQ, PTQ, and RRS. Reliable change indices were calculated by comparing the scores at T1, FU1, or FU2 to pretreatment scores. Hedges’ *g* effect sizes were calculated for the changes in mean scores from T1, FU1, or FU2 to pretreatment scores.

On average, levels of the SCS at T1, FU1, and FU2 were higher than pretreatment levels. The levels of TMQ, GCQ, DAAPGQ, PTQ, and RRS at T1, FU1, and FU2 were lower than pretreatment levels.

| **Table 1.** Self-report individual and mean scores of potential mechanisms of change before and after treatment (*n* = 9) | | | | | | | | | | |  |  |
| --- | --- | --- | --- | --- | --- | --- | --- | --- | --- | --- | --- | --- |
|  | Measurement occasion | ID numbers | | | | | | | | |  | Effect size compared with pre-treatment |
| Self-report | | ID2 | ID3 | ID4 | ID9 | ID11 | ID13 | ID15 | ID16 | ID17 | Mean (SD) | Hedges’ *g* |
| SCS | Pre-treatment | 93 | 87 | 76 | 111 | 98 | 90 | 139 | 111 | 127 | 103.53 (20.24) |  |
|  | T1 | 162* | 96 | 69 | 100 | 96 | 76 | 140 | 108 | 138 | 109.44 (31.06) | -0.22 |
|  | FU1 | 158* | 91 | 103* | 105 | 113 | 104 | 127 | 110 | 137 | 116.44 (20.70) | -0.60 |
|  | FU2 | 145* | 86 | 116* | missing | 92 | 96 | 140 | 115 | 140 | 166.25 (23.52) | -2.73 |
| TMQ | Pre-treatment | 22 | 22 | 24 | 17 | 24 | 30 | 32 | 37 | 21 | 25.44 (6.29) |  |
|  | T1 | 14* | 17 | 20 | 17 | 24 | 25 | 35 | 35 | 23 | 23.33 (7.53) | 0.29 |
|  | FU1 | 18 | 16 * | 20 | 17 | 15* | 19* | 35 | 37 | 18 | 21.67 (8.28) | 0.49 |
|  | FU2 | 17 | 20 | 16* | 29* | 24 | 27 | 33 | 35 | 19 | 24.44 (6.97) | 0.14 |
| GCQ | Pre-treatment | 23 | 20 | 38 | 7 | 28 | 35 | 25 | 65 | 22 | 29.22 (16.12) |  |
|  | T1 | 0* | 22 | 19* | 11 | 19 | 42 | 27 | 56 | 3* | 22.11 (17.91) | 0.40 |
|  | FU1 | 0* | 27 | 18* | 8 | 19 | 20* | 32 | 56 | 0* | 20.00 (17.46) | 0.52 |
|  | FU2 | 0* | 18 | 14* | 25* | 4* | 31 | 30 | 61 | 1* | 20.44 (19.31) | 0.47 |
| DAAPGQ | Pre-treatment | 10 | 17 | 10 | 9 | 27 | 19 | 6 | 28 | 33 | 17.67 (9.75) |  |
|  | T1 | 0 | 4* | 5 | 15 | 9* | 20 | 9 | 34 | 20* | 12.89 (10.54) | 0.45 |
|  | FU1 | 0 | 12 | 4 | 11 | 9* | 9 | 5 | 33 | 17* | 11.11 (9.58) | 0.65 |
|  | FU2 | 0 | 14 | 5 | 15 | 9* | 18 | 10 | 31 | 4* | 11.78 (9.22) | 0.59 |
| PTQ | Pre-treatment | 28 | 54 | 33 | 29 | 45 | 51 | 54 | 64 | 39 | 44.11 (12.63) |  |
|  | T1 | 18* | 47* | 35 | 39* | 43 | 51 | 43* | 57* | 33 | 40.67 (11.36) | 0.27 |
|  | FU1 | 19* | 56 | 21* | 37* | 15* | 34* | 47* | 55* | 20* | 33.78 (16.02) | 0.68 |
|  | FU2 | 17* | 46* | 28 | 50* | 33* | 42* | 41* | 59 | 20* | 37.33 (13.96) | 0.49 |
| RRS | Pre-treatment | 8 | 12 | 11 | 12 | 9 | 9 | 8 | 12 | 10 | 10.11 (1.69) |  |
|  | T1 | 6* | 11 | 11 | 9* | 10 | 9 | 7 | 11 | 7* | 9.00 (1.94) | 0.58 |
|  | FU1 | 5* | 10* | 9* | 12 | 6* | 6* | 8 | 13 | 7* | 8.44 (2.79) | 0.69 |
|  | FU2 | 5* | 11 | 14* | 12 | 8 | 6* | 8 | 10 * | 9 | 9.22 (2.86) | 0.36 |

*Note.* SCS = Self-Compassion Scale; TMQ = Trauma Memory Questionnaire; GCQ = Grief Cognitions Questionnaire; DAAPGQ = Depressive and Anxious Avoidance in Prolonged Grief Questionnaire; PTQ = Perseverative Thinking Questionnaire; RRS = Ruminative Response Scale; * *p* < .05.

**References**

1. Neff K. The development and validation of a scale to measure self-compassion. Self Identity. 2003;2(3):223–50.

2. Boelen PA. Variables mediating the linkage between loss centrality and postloss psychopathology. J Nerv Ment Dis. 2012;200(9):801–6.

3. Halligan SL, Michael T, Clark DM, Ehlers A. Posttraumatic stress disorder following assault: the role of cognitive processing, trauma memory, and appraisals. J Consult Clin Psychol. 2003;71(3):419–31.

4. Boelen PA, Lensvelt-Mulders GLM. Psychometric properties of the Grief Cognitions Questionnaire (GCQ). J Psychopathol Behav Assess. 2005;27(4):291–303.

5. Boelen PA, van den Bout J. Anxious and depressive avoidance and symptoms of prolonged grief, depression, and posttraumatic stress-disorder. Psychologica Belgica. 2010;50(1/2):49–68.

6. Ehring T, Zetsche U, Weidacker K, Wahl K, Schonfeld S, Ehlers A. The Perseverative Thinking Questionnaire (PTQ): Validation of a content-independent measure of repetitive negative thinking. J Behav Ther Exp Psychiatry. 2011;42(2):225–32.

7. Ehring T, Weidacker K, Emmelkamp PMG, Raes F. Validation of the Dutch version of the Perseverative Thinking Questionnaire (PTQ-NL). Eur J Psychol Assess. 2012;28(2):102–8.

8. Schoofs H, Hermans D, Raes F. Brooding and reflection as subtypes of rumination: Evidence from confirmatory factor analysis in nonclinical samples using the Dutch Ruminative Response Scale. J Psychopathol Behav Assess. 2010;32(4):609–17.

9. Treynor W, Gonzalez R, Nolen-Hoeksema S. Rumination reconsidered: A psychometric analysis. Cogn Ther Res. 2003;27(3):247–59.
