# Supplementary material for: Cognitive behavioural therapy and mindfulness for relatives of missing persons: a pilot study
Source: Pilot Feasibility Stud. 2019 Jul 20;5:93. doi: 10.1186/s40814-019-0472-z (PMC6642737; doi:10.1186/s40814-019-0472-z)
Supplement: Supplementary file 3 — Supplementary material C. (DOCX 38 kb) [file 40814_2019_472_MOESM3_ESM.docx]

**Additional file 3**

Figure 1 displays the average reductions (in percentages) in PCBD, MDD, and PTSD for completers in both conditions. These rates were computed by 100-((Average post-treatment score/average pre-treatment score)*100). In black, the reductions from pre-treatment to post-treatment for the immediate intervention group are displayed. In white, the reductions from pre-waiting to post-waiting for the waiting list controls are displayed. On average, the participants in the immediate intervention group report 25.6% to 41.5% reduction in PCBD, MDD, and PTSD levels, whereas the waiting list controls report 5.7% to 11.6% reduction in PCBD and MDD levels, respectively, and 132.9% increase in PTSD levels.

**Figure 1.** Percentages of reductions in mean PCBD, MDD, and PTSD levels for the immediate intervention (*n* = 5) and waiting list control condition (*n* = 4).


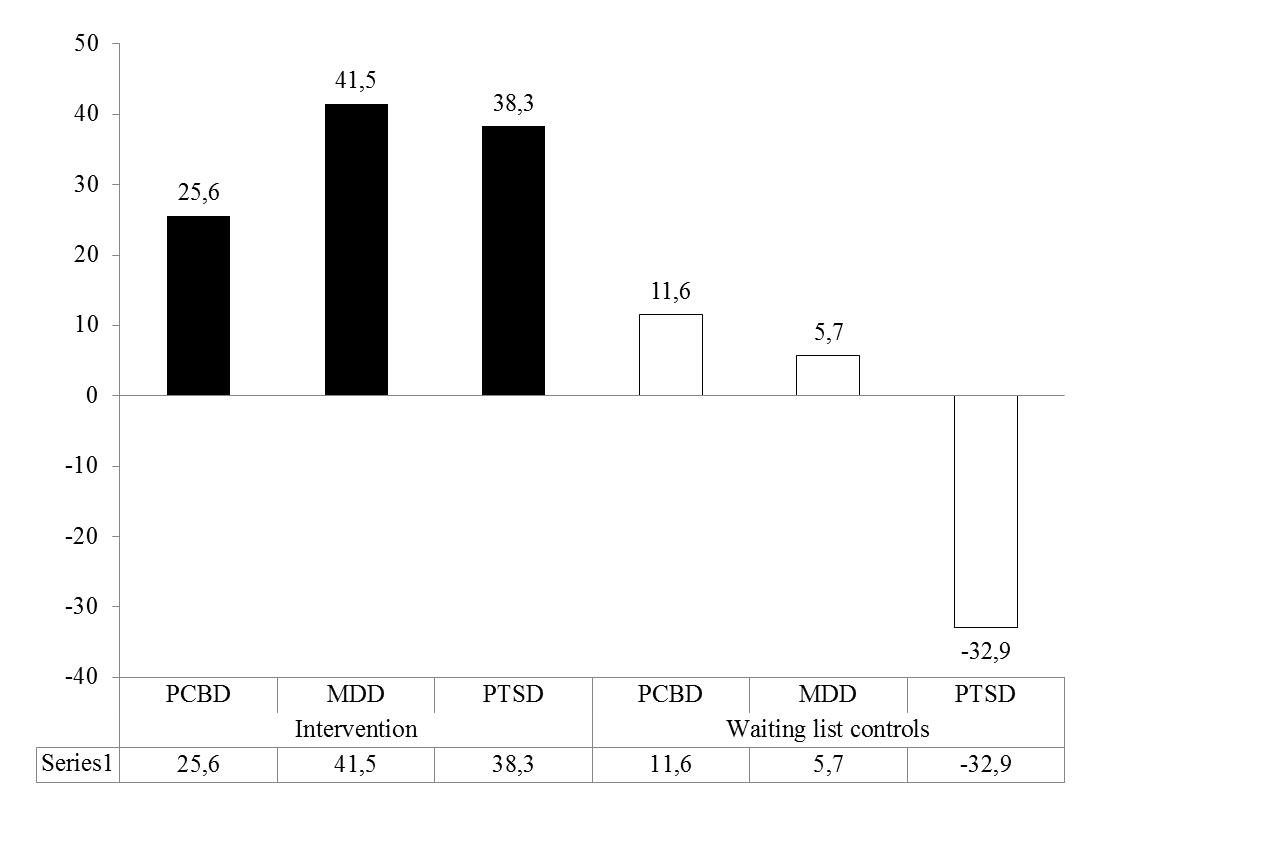


*Note.* PCBD = persistent complex bereavement disorder; MDD = major depressive disorder; PTSD = posttraumatic stress disorder.
